# The role of ApoE-mediated microglial lipid metabolism in brain aging and disease

**DOI:** 10.1097/IN9.0000000000000018

**Published:** 2023-01-23

**Authors:** Jui-Hung Jimmy Yen, I-Chen Ivorine Yu

**Affiliations:** 1 Department of Microbiology and Immunology, Indiana University School of Medicine, Indianapolis, IN, USA; 2 Department of Anatomy, Cell Biology, and Physiology, Indiana University School of Medicine, Fort Wayne, IN, USA

**Keywords:** microglia, lipid metabolism, apolipoprotein E

## Abstract

Microglia are a unique population of immune cells resident in the brain that integrate complex signals and dynamically change phenotypes in response to the brain microenvironment. In recent years, single-cell sequencing analyses have revealed profound cellular heterogeneity and context-specific transcriptional plasticity of microglia during brain development, aging, and disease. Emerging evidence suggests that microglia adapt phenotypic plasticity by flexibly reprogramming cellular metabolism to fulfill distinct immune functions. The control of lipid metabolism is central to the appropriate function and homeostasis of the brain. Microglial lipid metabolism regulated by apolipoprotein E (ApoE), a crucial lipid transporter in the brain, has emerged as a critical player in regulating neuroinflammation. The ApoE gene allelic variant, *ε4*, is associated with a greater risk for neurodegenerative diseases. In this review, we explore novel discoveries in microglial lipid metabolism mediated by ApoE. We elaborate on the functional impact of perturbed microglial lipid metabolism on the underlying pathogenesis of brain aging and disease.

## 1. Introduction

Microglia are the brain-resident innate immune cells that constantly monitor the brain microenvironment, initiate defense responses against insults, and exert multifaceted functions during brain development, aging, or disease ^[1]^. Microglia show distinct transcriptional programs throughout the lifespan and exhibit heterogeneous characteristics among subpopulations ^[2–4]^. Although microglia are transcriptionally different from the bone marrow-derived macrophages ^[5,6]^, microglia share similarities with peripheral immune cells in the aspects of metabolic adaptation to modulate their immunophenotypes ^[7]^. Peripheral immune cells, such as macrophages, dendritic cells, and T lymphocytes, process profound flexibility to change cellular metabolism under inflammatory conditions and orchestrate their cellular functions ^[8,9]^. Resting macrophages rely on the tricarboxylic acid (TCA) cycle in the mitochondria to drive oxidative phosphorylation (OXPHOS) and generate high amounts of adenosine triphosphate (ATP) from various nutrient sources. Upon stimulated by lipopolysaccharide (LPS) or interferon γ (IFN-γ), macrophages increase their glycolytic demands as a step toward proinflammatory activation, which yields a much lower amount of ATP to balance the needs of macromolecules syntheses ^[10,11]^. The emerging field of immunometabolism research on the peripheral immune cells has scaffolded the regulatory nexus of cellular metabolism for the study of microglial biology ^[12]^.

Growing evidence indicates that microglia can utilize various metabolic profiles to modify phenotypes similarly to peripheral macrophages. Here, we review research highlights in microglial immunometabolism and present emerging findings on the diverse role of lipids and the lipid carrier, apolipoprotein E (ApoE), in regulating the microglial metabolism and function.

## 2. Immunometabolism of microglia

Brain energy metabolism is tightly controlled to support the highly metabolic demands of neuronal activity and maintain proper function ^[13]^. Many neurological disorders are often associated with the dysregulation of brain energy metabolism ^[14,15]^. As microglia constantly scan the brain microenvironment during surveillance, it is speculated that microglia require high-energy demand and metabolic flexibility to make adaptive changes dynamically. Transcriptional studies indicate that microglia express the full spectrum of genes in major cellular metabolic pathways, such as glycolysis and OXPHOS ^[7,16]^. These studies suggest the capacity of microglia to utilize bioenergetic switches to regulate their cellular functions.

Homeostatic microglia are thought to rely on OXPHOS for ATP production, whereas activated microglia display a metabolic switch toward glycolysis. Activating microglial-like BV-2 cells with LPS and IFN-γ induces altered mitochondrial OXPHOS respiration ^[17,18]^ and shifts the cellular metabolism toward glycolysis ^[19,20]^. This promotes the production of proinflammatory cytokines. Using real-time flux assays to measure the extracellular acidity and oxygen consumption, several studies report that microglia increase glycolytic flux responding to the proinflammatory stimulation or retain more mitochondrial respiration capacity under the stimulus of anti-inflammatory cytokines, such as IL-4 ^[21–23]^. In support of metabolic reprogramming, manipulating genes involved in glycolytic pathways controls microglial inflammatory phenotypes. Induction of the key glycolytic enzyme, hexokinase 2 (HK2), triggers the accumulation of acetyl-coenzyme A, which modifies the acetylation on histone proteins and induces the transcription of proinflammatory IL-1β. In contrast, knocking down HK2 results in the suppression of IL-1β expression in microglia ^[24]^. Increased expression of another enzyme along the glycolytic pathway, 6-phosphofructo-2-kinase/fructose-2,6-biphosphatase (PFKFB) 3, is found to confer glycolytic and inflammatory activation of microglia in Alzheimer’s disease state ^[25,26]^. Several studies have also highlighted the role of metabolic intermediates in regulating the phenotypic polarization of microglia. The glycolytic byproduct, lactate, can incite a novel epigenetic modification to regulate gene transcription of glycolytic enzymes, setting up a positive-feedforward loop to enhance inflammatory activation of microglia ^[27]^. By contrast, the mitochondrial OXPHOS byproduct, itaconate, exerts anti-inflammatory polarizing effects on microglia under different neuroinflammatory conditions ^[28,29]^.

Besides glucose utilization, microglia are metabolically flexible. Microglia can utilize glutamine as a bioenergetic source under hypoglycemic conditions ^[30,31]^. Microglia also metabolize other amino acids to generate ATP and facilitate phenotypic switches. Culturing microglial cells under high serine/glycine conditions promotes the neurotoxicity phenotype ^[32]^. In contrast, feeding microglia with high contents of branched-chain amino acids, such as valine, leucine, and isoleucine, enhances IL-10 mRNA expression and promotes the phagocytosis stimulated by LPS ^[33]^. Modulating tryptophan metabolism through the kynurenine pathway dampens tumor necrosis factor α (TNFα) expression in microglia and the extracellular nitrite accumulation following LPS stimulation ^[34,35]^. These studies support the concept that microglial function is regulated by complex but rapid reprogramming of the cellular metabolic pathways.

Recent single-cell sequencing analyses showed that glycolytic genes, such as lactate dehydrogenase A (*Ldha*), are upregulated in proinflammatory human microglia and some microglial subpopulations during early development stage in the mouse brain ^[2,36]^. These transcriptomic analyses highlight the notion that microglia exploit different metabolic signatures to reshape their phenotypes under physiological and pathological conditions. The genes involved in the cellular metabolic pathways are differentially enriched among microglial subpopulations, in which CD11c+ early postnatal microglia show the exclusive expression of genes involved in fatty acid oxidation, proline, and glutathione metabolisms ^[6]^. By contrast, genes involved in mitochondrial OXPHOS respiration are exhibited in human disease–associated microglia. Further investigations are warranted to shed mechanistic insights into the immunometabolism of microglia in controlling neuroinflammation in brain aging and disease.

## 3. Microglial lipid metabolism

Lipids are essential regulators for membrane structure, cellular bioenergetics, and signal transduction in the brain ^[37]^. Brain lipids are enriched with phospholipids, sphingolipids, ceramides, and cholesterols to meet the specialized function of the brain ^[38]^. Brain lipid levels are tightly regulated by de novo synthesis and intercellular exchanges among different brain cells. Abnormal lipid homeostasis within glial cells is likely to alter neuron-glia interplay and the metabolic integrity of neurons, leading to accelerated neurodegeneration ^[39]^. Appropriate lipid transfer between neurons and glia via lipid transporters, such as ApoE, is essential for maintaining neuronal health.

Lipids are emerging as key players in immune cell homeostasis through their various distributions in the cell. The distinct microdomains in the plasma membrane, lipid raft, can initiate receptor engagement and signaling transductions ^[40,41]^. In contrast, intracellular lipids, such as mitochondrial lipids ^[42]^ or signaling messengers ^[43]^, can regulate immune responses through metabolic pathways. These studies in the peripheral immune cells suggest that lipid metabolism also plays an essential role in regulating microglial state and function. For instance, polyunsaturated fatty acids (PUFAs), such as *n*-3 series, docosahexaenoic acid (DHA), and eicosatetraenoic acid (EPA), can polarize microglia toward phagocytic phenotype and decrease inflammatory markers via metabolic reprogramming ^[44–46]^. In the following sections, we will discuss ApoE-mediated lipid metabolism to regulate microglial function.

### 3.1 Lipoprotein metabolism in microglia

Microglia express numerous receptors to monitor and sense alterations in the brain microenvironment ^[47]^. These receptors mediate lipid sensing, uptake, and signaling to regulate microglial function. For instance, the triggering receptor expressed on myeloid cells 2 (TREM2) binds various lipid ligands, such as phospholipids and myelin debris, to incite the phagocytosis of damage-associated lipids by microglia ^[48,49]^. TREM2 also recognizes ApoE, the brain’s predominant carrier of cholesterol transport.

Cholesterol has many essential functions within the brain cells. Brain cholesterol accounts for 23% of the body’s total cholesterol content ^[50]^, and is mainly synthesized by astrocytes to supply sterol lipids to neurons ^[51]^. Besides astrocytes, oligodendrocytes synthesize substantial amounts of cholesterol to promote myelin formation, making myelin the largest storage of the brain’s cholesterol ^[52]^. ApoE generated from astrocytes forms ApoE/lipid particles to shuttle cholesterols or its derived lipids in or out of neurons for axonal growth, synapse formation, or prevention of lipid toxicity in neurons ^[53,54]^. Recent single-cell RNA sequencing studies have found that microglia upregulate, whereas astrocytes downregulate the ApoE expression in human brains with Alzheimer’s disease pathology ^[55]^. Consistent findings are reported in different mouse models of Alzheimer’s disease ^[56–59]^. Although the increased expression of ApoE in the microglia subpopulations might reflect a response to increased intracellular cholesterols, the ApoE particles derived from microglia are reported to have smaller sizes, indicating an altered lipidation state ^[60]^. These findings suggest that cell type-specific expression of ApoE might modulate the state of lipidation or post-translational modifications on ApoE. Whether the shift of ApoE secretion from an astrocytic source to a microglial origin contributes to different biological functions remains to be determined to elucidate the impact of microglial-derived ApoE in the pathogenesis of Alzheimer’s disease.

Cholesterol clearance is an essential function of microglia to maintain homeostatic cell-to-cell lipid distribution in the brain. ApoE mediates cholesterol clearance through binding to various receptors expressed on microglia, such as low-density lipoprotein receptor (LDLR), LDL receptor-related protein 1 (LRP1), and TREM2 ^[49,59,61,62]^. The binding of ApoE particles with surface receptors initiates endocytosis, in which microglia metabolize cholesterols into hydroxycholesterols, phospholipids, and free fatty acids. These lipids subsequently activate lipid-responsive receptors, such as the liver X receptor (LXR) and the peroxisome proliferator-activated receptor (PPAR), to regulate the expression of cellular efflux proteins, ApoE, and ATP-binding cassette (ABC) transporters, ABCA1 and ABCG1 ^[63,64]^. The ABCA1 is essential for incorporating lipids into the ApoE particles and controlling the ApoE lipidation states ^[65]^. These proteins involved in the cellular efflux machinery are critical for intracellular lipid homeostasis. When less efficiently removing cholesterol-derived lipids, lipid droplets (LDs) build-up exhibits as an adaptive response to sequester excessive lipids inside microglia. However, the accumulated LDs have been recognized as dynamic organelles to regulate microglial phenotypes and functions ^[66–68]^. LD-accumulating microglia are identified as a new state of microglia during brain aging, which exhibits lysosomal membrane rupture and activated inflammasomes, indicating the proinflammatory activation of LD-rich microglia ^[69]^. The LD-accumulating microglia in aging brains also show impaired phagocytosis and excessive production of proinflammatory cytokines ^[70]^. Under the demyelinating state, defective cholesterol clearance in microglia shifts the microglial phenotype toward proinflammatory activation ^[71]^. In a chimeric mouse model of Alzheimer’s disease, the human induced pluripotent stem cell (iPSC)-derived microglia are reported to be enriched with LDs and resemble human atherosclerotic foam cells ^[72]^. These findings suggest a broader spectrum of altered lipid metabolism observed in microglia during brain aging and various disease states. Further understanding the composition, biogenesis, and dynamics of LDs in microglia and how these LDs influence the capacity of mitochondrial OXPHOS respiration of microglia in the context of different pathological conditions will be important research questions to pursue.

Modulating ApoE has been shown to change the immunophenotypes of microglia profoundly. Increasing ApoE expression or activating the ApoE pathway by a pharmacological approach abrogates the function of microglia to suppress T cell proliferation, suggesting an impaired function of microglia to regulate immunotolerance ^[58]^. These studies also indicate the role of ApoE in microglia to control the potentially harmful self-reactive T cells under disease conditions. On the contrary, deleting ApoE in mice results in the accumulation of cholesteryl ester in microglia ^[49,69]^. It induces subpopulations of microglia that show gene signatures of activation and upregulated genes encoding glycolytic enzymes, such as *Ldha* ^[59]^. It is speculated that the accumulation of cholesterol esters and LDs might dampen mitochondrial OXPHOS, leading to the shift of metabolism toward glycolysis and inflammatory activation of microglia. These findings suggest that disturbed lipid metabolism has emerged as the pathogenicity of microglial dysfunction in brain aging and diseases.

### 3.2 ApoE4-mediated microglial lipid metabolism and inflammation: implications in Alzheimer’s disease and related neurodegenerative disorders

In humans, ApoE exists three isoforms, ApoE2, ApoE3, and ApoE4, encoded by allelic variants at the *Apoe* gene locus on chromosome 19 ^[73]^. Among ApoE’s allelic variants (*ε2, ε3, ε4*), the *ε4* allele, encoding the ApoE4 isoform, carries the most significant risk for the development of the late-onset Alzheimer’s disease ^[74,75]^. ApoE2 and ApoE4 isoforms differ from ApoE3 by a single amino acid substitution at positions 112 and 158 (ApoE2: Cys112 and Cys158, ApoE3: Cys112 and Arg158, and ApoE4: Arg112 and Arg158). Several structural studies suggest that the Cys to Arg amino acid substitution changes the structure features of protein domains, resulting in differential binding affinities to lipids and receptors among three ApoE isoforms ^[76–79]^. Differences in biochemical properties of ApoE isoforms potentially influence the receptor-mediated clearance of Aβ ^[80–83]^. In addition, the biochemical differences among ApoE isoforms can affect the presentation of protein domains or the aggregation propensity of proteins, which results in discrepancies in the ApoE levels detected. Previous studies report that ApoE2 displays the highest levels, followed by ApoE3, then ApoE4, in the brain or cerebrospinal fluid (CSF) from the human or human ApoE-expressing mouse models ^[84–87]^. Although other studies report no differences in the ApoE levels among three isoforms ^[88,89]^, a recent study using new human ApoE knock-in mouse lines supports the reduction of brain ApoE4, compared to ApoE3, in the detergent-soluble aggregated fractions ^[90]^. These findings suggest that the isoform-dependent risk (ApoE4 > ApoE3 > ApoE2) to late-onset Alzheimer’s disease may be contributed by the differences in biochemical properties of ApoE isoforms and the subsequent cellular cascades that ApoE regulates.

The structural differences among ApoE isoforms also lead to differential binding preferences to lipid particles. In the plasma, ApoE4 preferentially binds to large, triglyceride-rich very low-density lipoprotein (VLDL) particles, whereas ApoE3 and ApoE2 tend to associate with small, phospholipid-rich high-density lipoprotein (HDL) particles ^[73,91]^. However, in CSF and the brain, the ApoE–lipid complex exhibits different size features (ApoE2 > ApoE3 > ApoE4), depending on the lipidation status of ApoE proteins ^[92,93]^. Astrocyte-derived ApoE2 is found to be hyperlipidated compared to ApoE3 and ApoE4 ^[92,94]^, leading to the ApoE2 more efficiently promoting the efflux of cholesterol-derived lipids from cells. The less efficient cholesterol efflux mediated by ApoE4 might lead to the accumulation of intracellular LDs. This notion is supported by recent studies reporting more significant LDs formation in ApoE4-expressing astrocytes ^[95–97]^. Besides astrocytes, ApoE4-expressing microglia exhibit more intracellular LDs accumulation compared to ApoE3-expressing microglia ^[97]^. Here, we will focus on the role of the ApoE4 isoform involved in the alterations of lipid metabolism and function in microglia.

The mechanisms of ApoE4 contributing to the pathogenesis of Alzheimer’s disease are linked to neuroinflammation. High levels of proinflammatory mediators are associated with the ApoE4-expressing microglial cultures upon stimulated with LPS ^[98–100]^ or Aβ ^[101,102]^. ApoE4 exhibits a reduced ability to suppress inflammatory stimulus compared to ApoE3 ^[58,103]^. These findings suggest that the ApoE4-expressing microglia are predisposed toward proinflammatory states. Under lipid stress, oxidized hydroxycholesterol amplifies the proinflammatory IL-1β signaling in ApoE4-expressing microglia, supporting an intriguing convergence of lipid metabolism and inflammation mediated by ApoE4 ^[104]^.

Recently, human iPSCs-derived microglia-like cells have emerged as a powerful tool to investigate the mechanisms of ApoE4 variants in human microglia ^[105]^. The ApoE4-expressing microglia exhibit inflammatory gene signatures, accompanied by less Aβ uptake and clearance ^[106]^. In another iPSCs-derived microglia study, ApoE4 expression drives de novo cholesterol synthesis via the up-regulation of sterol regulatory element binding protein 2 (SREBP2). It impairs cholesterol trafficking in microglia through lysosomal sequestration of free cholesterols ^[107]^. The altered cholesterol metabolism significantly reduces the capacity of phagocytosis in ApoE4-expressing microglia to uptake myelin debris, resembling the cellular state as shown in aging microglia ^[70]^. A recent report suggests that the expression of ApoE4 downregulates genes involved in mitochondrial OXPHOS respiration and promotes genes required for lipogenesis, resulting in LDs accumulation in microglia ^[108]^. The reduction of cholesterol uptake in ApoE4-expressing microglia leads to the extracellular accumulation of cholesterol, which is incorporated into the cell membrane of neurons and potentiates the activity of the lipid-gated potassium ion channels. Modulating intracellular LDs content through pharmacological inhibition of acyl-CoA synthetase 1 (ACSL1) is found to restore the purinergic signaling and sustain the homeostatic states of microglia to support neuronal networks ^[108]^. Together, these findings suggest that the convergence of lipid metabolism and inflammatory activation triggers the dysfunction of cellular pathways in microglia, resulting in disrupted neuron–microglia interactions and, eventually, neurodegeneration.

## 4. Future perspective

Our understanding of microglial metabolism in the brain has advanced significantly over the past few years because of the burning field of immunometabolism. New perspectives on the role of lipid metabolism in regulating microglia have recently emerged. However, some questions remain unsolved, and the literature survey raises new research questions.

- The functional impact? The two most significant risk factors of Alzheimer’s disease and related neurodegenerative disorders, aging and ApoE, converge to microglia and neuroinflammation. The inflammation-aging (inflammaging) axis has set a step for microglia to undergo senescence as other immune cells during aging ^[109,110]^. Various lipid classes have been linked to the senescence processes. For instance, phospholipids and phospholipases are involved in stress-induced senescence, and senescent cells accumulate LDs ^[111]^. Diacylglycerols (DAGs) and triacylglycerols (TAGs) are the major glycolipids involved in LDs and are coupled to cellular metabolism to regulate their interactions with other organelles ^[112]^. Sphingolipids are highly bioactive lipids involved in cellular senescence ^[113]^. It is critical to dissect how ApoE reshapes the LDs or sphingolipids to promote proinflammatory activation of microglia, which might uncover an unrecognized mechanism of microglial lipid metabolism contributing to immunosenescence of microglia in brain aging and disease.
- The cell-autonomous effects of ApoE on microglia? Positioning signaling receptors, such as toll-like receptor 4 (TLR4), into the cholesterol-rich microdomain, lipid raft, on the plasma membrane is critical to initiating proinflammatory activation of macrophages ^[114]^. The excessive extracellular cholesterols are found to be incorporated into neurons, forming lipid raft microdomains to control lipid-gated G-protein coupled ion channels ^[108]^. Whether the extracellular or intracellular cholesterols are incorporated into plasma membranes as lipid rafts in ApoE4-expressing microglia will require further investigation.
- The in vivo validation? Many studies of microglial immunometabolism rely on primary microglial cultures from animal models. Employing new chemical imaging techniques, such as Coherent Anti-Stokes Raman Scattering (CARS) microscopy, to analyze temporal or spatial alterations in LDs will be important to bridge the gap in our understanding of microglial lipid metabolism ^[115,116]^. Moreover, the evolving mass spectrometry techniques can allow us to apply the spatiotemporal characterizations of lipid signaling via mass spectrometry imaging ^[117,118]^ or use lipidomics approaches to profile highly diverse lipid species in microglia. These lipidomics approaches can systemically integrate with data obtained from transcriptomics, proteomics, or metabolomics studies to elucidate the role of lipid and lipid metabolism in microglial function ^[119]^.

All these research questions will let us dissect further the role of metabolism influencing microglial function and how it shapes microglia participating in the pathogenesis of brain aging and disease.

## 5. Conclusions

Neuroinflammation is a central element involved in the pathogenesis of brain aging and various neurological disorders, particularly Alzheimer’s and related neurodegenerative diseases. With emerging techniques and experimental strategies, we are only beginning to untangle the role of lipid metabolism controlling microglial functions and the impact of microglial lipid dysregulation on the neuron-glia network in the context of disease. A better understanding of metabolic regulation in microglia and future investigations are required to uncover promising functional biomarkers or new immune-therapeutic avenues for various neurodegenerative disorders.

## Author contributions

J-HJY wrote the manuscript. I-CIY wrote, reviewed, and edited the manuscript.

## Conflicts of interest

The authors declare that they have no conflict of interest.

## Funding

This work was supported by grant R21AG070971 “Dynamic immune cell landscape in late-onset Alzheimer’s disease: role of ApoE-mediated microglial lipid metabolism”.
